# Relationship of serum CEA levels to tumour size and CEA content in nude mice bearing colonic-tumour xenografts.

**DOI:** 10.1038/bjc.1981.195

**Published:** 1981-09

**Authors:** J. C. Lewis, P. A. Keep

## Abstract

**Images:**


*Br. J. Cancer* (1981) **44**, 381

### RELATIONSHIP OF SERUM CEA LEVELS TO TUMOUR SIZE AND CEA CONTENT IN NUDE MICE BEARING COLONIC-TUMOUR XENOGRAFTS

J. C. M. LEWIS AND P. A. KEEP

*From the Department of Medical Oncology, Charing Cross Hospital, Fulham Palace Road, London*

Received 5 March 1981 Accepted 7 May 1981

**Summary.—The relationship of serum carcinoembryonic antigen (CEA) levels to tumour size and antigen content was studied in nude mice bearing well differentiated, mucinous human colonic-tumour xenografts. Blood samples were taken from normal nude mice and others bearing xenografts, whose size had been calculated from *in vivo* measurements; saline and KCl extracts were made of a proportion of these tumours. Sera and tissue extracts were assayed for CEA activity by double-antibody radioimmunoassay. Extracts were also made from the livers and spleens of tumour-bearing and normal nude mice.**

**All normal sera and 78% of sera from tumour-bearing animals had CEA values <11·4 ng/ml. No clear correlation was found between serum CEA levels >11·4 ng/ml and tumour size or weight, or between serum CEA and tumour CEA concentrations or total CEA burden. The concentration of CEA in those tumours tested varied from 1 to 22 μg/g.**

**Our results confirm and extend the conclusions reached by others (Stragand *et al.*, 1980) studying the significance of serum CEA levels with xenograft model systems. The complexity of factors contributing to circulating CEA is discussed in the light of our findings.**

DURING RECENT YEARS much attention has been focused on the relationship between circulating carcinoembryonic antigen (CEA) levels and various aspects of colorectal tumour biology (*e.g.* size, degree of differentiation, extent of metastatic spread). However, it is difficult to draw many firm conclusions from the considerable volume of published data. Some studies have shown that circulating CEA levels may not be directly related to tumour size (Reynoso *et al.*, 1972; Holyoke *et al.*, 1972) although it must be said that measurement of tumour size in the patient is extremely difficult. A lack of correlation has also been reported between circulating CEA levels and the degree of tumour differentiation (Dhar *et al.*, 1975; Bivins *et al.*, 1975) or tumour CEA content (Gallo *et al.*, 1977; Khoo *et al.*, 1973; Goldenberg *et al.*, 1976*b*; McClendon *et al.*, 1977). Several authors, however, have suggested that circulating CEA levels more accurately reflect the general extent of disease (Dhar *et al.*, 1975; Zamcheck *et al.*, 1975; Breuer *et al.*, 1980) and that this may, in part, be a result of the degree of necrosis and vascular invasion within the tumour (Bivins *et al.*, 1975; Zamcheck *et al.*, 1975; Breuer *et al.*, 1980).

It is surprising, therefore, that very few studies have been published using *in vivo* model systems—especially colonic-tumour xenografts. One widely quoted study of nude mice bearing well differentiated colonic-tumour xenografts proposed that serum CEA levels were indeed directly proportional to tumour size (Miwa *et al.*, 1976, 1977; Kubota *et al.* 1978). However, little reliance, if any, can be placed on this conclusion, given the low number of samples (5). Other authors mentioning a similar relationship have also based their findings on very low sample numbers (Mach *et al.*, 1974). In contrast, a much larger study (Stragand *et al.*, 1980), using nude mice bearing "LoVo" colonic-tumour xenografts, demonstrated no correlation between tumour size and serum CEA. Similar findings have been reported for immune-deprived mice bearing xenografts of breast carcinoma (Davies & Steel, 1978). It has also been found that blood AFP levels in immune-deprived mice bearing human malignant teratoma xenografts did not correlate well with tumour size (Raghavan *et al.*, 1980).

The present work was conducted to assess the relationship between circulating CEA levels and tumour size, weight, CEA concentration and total tumour CEA burden in nude mice bearing a well differentiated mucinous colonic-tumour xenograft (P116), and to assess the variation of CEA concentration within an apparently stable xenograft line. In making extracts of several tissues from both tumour-bearing and normal nude mice, it was our intention to define extra-tumour sites of CEA retention in tumour-bearing animals, and determine whether normal tissues of the nude mouse express cross-reacting antigens undetectable in serum assays.

### MATERIALS AND METHODS

Outbred female athymic nude (*nu/nu*) mice were maintained in a clean conventional environment and fed irradiated diet and chlorinated ($\sim 1/10^6$ chlorine) water *ad libitum*. Tumour material was implanted s.c. under ether at 10 weeks of age, and normal control animals used at 20 weeks.

The P116 xenograft line maintained at Charing Cross Hospital was originally established in immune-deprived mice (Cobb, 1973). Over serial passage in nude mice the histological appearance has varied little and consists of moderately well differentiated adenocarcinoma cells forming clumps, gland-like structures and single layers with abundant

FIG. 1.—Photomicrograph of P116 xenograft of the 5th passage. H. & E. × 65.

extracellular mucin secretion (Fig. 1). Central necrosis is a consistent feature. The tumours in nude mice are surrounded by and invested peripherally with a fibrous stroma containing typically a moderate and patchy chronic inflammatory infiltrate. Metastasis has not been seen in tumour-bearing animals.

Fifty-five mice bearing xenografts of the 5th and 6th passages were bled under ether anaesthesia from the retro-orbital venous sinus at various times after xenografting. Blood samples were similarly taken from 18 control animals. Serum was separated by centrifugation after overnight clotting at room temperature. Tumours were measured in 3 dimensions with calipers (L, W & H), tumour volumes estimated as LWH/2 (Looney *et al.*, 1973) and weights estimated from previously constructed calibration curves. Estimated tumour weights ranged from 0·02 g to 7·20 g with a mean of 1·08 g.

Eleven of the 55 tumour-bearing mice and 5 control mice were killed by cervical dislocation after bleeding, and tumours, livers and spleens removed. All organs were weighed, and a small sample of each tumour fixed in 10% formalin for subsequent histological examination. Tumours were then reweighed. Whole tumour weights ranged from 0·16 to 7·14 g (mean 1·61 g).

Tissue samples were homogenized in physiological saline and the homogenates centrifuged at 75,000 *g* for 30 min. Supernatants were dialysed against twice-daily changes of tap water at 4°C for 72 h, and concentrated to ~5 ml by ultrafiltration. Samples were centrifuged for a second time at 75,000 *g*, 30 min, and the supernatants stored at −20°C with 0·02% sodium azide, after recording their volumes. These samples were designated saline extracts.

Homogenate sediments produced by the first ultracentrifugation were washed once with physiological saline and extracted for 72 h with an excess of 3M KCl. Extracts were centrifuged, dialysed, concentrated, re-centrifuged and stored as above. These samples were designated KCl extracts.

CEA levels in tumour and normal-tissue extracts and serum samples were measured by double-antibody radioimmunoassay. CEA isolated from hepatic metastases of colonic tumours by perchloric acid extraction and purification on Sepharose 6B and Sephadex G-200 (Keep *et al.*, 1978) (Preparation M12) was used as standard (8 doubling dilutions of top standard 500 ng/ml CEA). The CEA preparation used as label was extracted as above and purified further on Con A-Sepharose (Keep *et al.*, 1978) and designated CEA-2B. Iodination was carried out by a modification of the Chloramine T technique (Greenwood *et al.*, 1963) yielding a specific activity of 179 μCi/μg protein.

The primary antiserum (Pk1G (D2)) was raised in a goat to CEA-2B, then absorbed with normal human plasma and perchloric acid extracts of normal human spleen, colon and liver in immunoabsorbent columns. The absorption was monitored by rocket immunoelectrophoresis of the absorbed antiserum against a 20mg protein/ml solution of each normal human tissue. The absence of rockets was taken to indicate lack of cross-reaction. Precipitation of the CEA–anti-CEA complexes was achieved with second antibody (BW402 horse anti-(goat+sheep)).

For the tumour and normal-tissue extracts (buffer-based system) 10 zero-antigen tubes (maximum binding of label) were set up with buffer (0·05M phosphate, pH 6, containing BSA (0·1% w/v) and EDTA (3·4 mM); 200 μl), primary antiserum (dilution 1/4400; 50 μl) and CEA label (80,000 ct/min; 50 μl). Into 10 nonspecific binding tubes were placed buffer (250 μl) and label (50 μl) only. The standards, and samples at appropriate dilutions in buffer were set up in triplicate containing standard/sample (100 μl), buffer (100 μl), primary antiserum (50 μl) and label (50 μl). Incubation was allowed to proceed for 16 h at 37°C and then second antibody (dilution 1/40; 50 μl) added. After a further incubation at 37°C for 8 h the bound fraction was obtained. Filtration, counting and analysis was performed on the Kemtek 3000 automated radioimmunoassay system.

Measurement of serum samples was essentially as described above, except that all samples (in triplicate) were assayed directly at dilution 1/4. Insufficient serum was available to assay neat serum in triplicate. Buffer (100 μl) was added to the samples and normal nude mouse serum (*nu*/*nu*) at dilution 1/4 (100 μl) added to the zero-antigen, nonspecific binding and standard tubes.

Doubling dilutions of each sample were measured to ensure a parallel response to the standard line. The interassay coefficient of variation was 13%. Since the sensitivity of the radioimmunoassay was 2·85 ng/ml and the serum samples were all measured at dilution 1/4, the lower cut-off point was set at a CEA value of 11·4 ng/ml. This was not taken to represent the upper limit of serum CEA in normal nude mice but rather as the lower limit of detection in the diluted serum samples, necessitated by our insistence upon triplicated assays.

Tumour samples fixed in formalin were embedded in paraffin wax and 5$\mu$M sections cut and stained with haematoxylin and eosin by conventional methods.

### RESULTS

The histological appearance of all tumour samples was typical of the xenograft line (as described in Materials and Methods).

All sera from control animals and 43 of the 55 sera from tumour bearers had CEA values < 11·4 ng/ml. The remaining 12 showed values ranging from 13·9 to 47·8 ng/ml (and tumour weights 0·22–7·14 g). No clear correlation was found between the serum CEA values and tumour size or weight (Fig. 2). However, all

FIG. 2.—Serum carcinoembryonic antigen levels *vs* tumour weight in xenograft-bearing nude mice. (55 points, 12 > 11·4 ng.)

animals bearing tumours > 2·15 g had raised serum CEA values. The coefficient of correlation ($r$) between serum CEA (when > 11·4 ng/ml) and tumour weight was 0·46.

Tumour CEA concentration varied between ~1 and 22 $\mu$g/g wet weight of tumour (total CEA extracted by the two methods) and, in the 11 animals studied, neither tumour CEA concentration nor

TABLE

| Tumour | Tumour weight (g) | Tumour (CEA) ($\mu$g/g) | Total tumour CEA burden ($\mu$g) | Serum CEA (ng/ml) |
|---|---|---|---|---|
| 1 | 0·16 | 1·16 | 0·17 | < 11·4 |
| 2 | 0·22 | 20·43 | 4·58 | 16·3 |
| 3 | 0·39 | 13·62 | 5·35 | < 11·4 |
| 4 | 0·79 | 22·02 | 17·42 | < 11·4 |
| 5 | 1·10 | 14·43 | 15·89 | < 11·4 |
| 6 | 1·16 | 21·05 | 24·44 | < 11·4 |
| 7 | 1·16 | 4·86 | 5·65 | < 11·4 |
| 8 | 1·50 | 2·90 | 4·34 | < 11·4 |
| 9 | 1·96 | 3·24 | 6·35 | < 11·4 |
| 10 | 2·14 | 12·18 | 26·08 | < 11·4 |
| 11 | 7·14 | 13·69 | 97·74 | 47·8 |

total tumour CEA burden (CEA concentration × tumour weight) appeared to correlate with serum CEA values (Table). However, we feel that firm conclusions on this point cannot be drawn from the data presented owing to the low number of serum CEA values > 11·4 ng/ml in the group of animals from which extracts were made (2/11). There was no correlation between tumour CEA concentration and tumour weight or size ($r = -0·01$).

Liver and spleen extracts from control animals were negative for CEA. CEA was detected in KCl extracts of the livers of 2 tumour-bearers only (Tumours 6 & 11 in the table, with 0·08 $\mu$g CEA/g liver and 0·16 $\mu$g CEA/g liver respectively) and in none of the saline extracts. Saline extracts of spleens from 2 tumour-bearers were positive for CEA (Tumours 10 and 11 in the table, with 0·25 $\mu$g CEA/g spleen and 0·85 $\mu$g CEA/g spleen respectively), and KCl extracts were all negative. The presence of CEA in the non-tumoral organs of tumour-bearing mice appeared to be correlated with high total tumour-CEA burden, but not with high tumour-CEA concentration, nor with high serum CEA. However, with so few positive values for these tissues, firm conclusions cannot be made from the data.

### DISCUSSION

Our study demonstrates a lack of correlation between serum CEA values and tumour weight or size in nude mice bearing P116 colonic-tumour xenografts. With an assay cut-off of 11·4 ng/ml, small increases in serum CEA of tumour-bearing nudes may have gone undetected, artificially reducing the proportion of "positive" sera reported in our sample. It is possible, therefore, that the correlation coefficient between serum CEA and tumour weight may be greater than the 0·46 quoted. However, it remains apparent from the spread of serum CEA values shown in Fig. 2 that these values cannot be used to predict tumour weight or size, even if a proportion of sera at $<11{\cdot}4$ ng CEA/ml were in fact to range between 0 and 11·4 ng according to tumour weight. It is further suggested that in this model neither the tumour CEA concentration nor total tumour CEA burden correlate with serum values. These results are in agreement with those of Stragand *et al.* (1980). It is possible, though, that CEA in serum samples could be undetected in a direct radioimmunoassay owing to the presence of nude mouse anti-CEA immunoglobulins formed in response to the xenograft. Anti-CEA (IgM) has been detected in hamsters bearing GW39 colonic tumour xenografts (Primus *et al.*, 1973*b*; Primus *et al.*, 1976) and, although the humoral immune responses of nude mice are severely impaired by the lack of functional T cells, it is conceivable that IgM and/or IgG anti-CEA could be elaborated to what is, in effect, a depot preparation of CEA.

Perhaps of more importance, the present work also demonstrates the wide range (22-fold) of CEA concentration to be found in examples of a single, apparently stable, xenograft line, and that this variation is not simply a function of tumour size. Whilst there is plenty of evidence for the widely varying concentrations of CEA in human colorectal carcinomas (Dyce & Haverback, 1974; Sharkey *et al.*, 1977; Khoo *et al.*, 1973; Warner *et al.*, 1973) and that differences may exist in regional CEA concentration within a single tumour (Wagener & Breuer, 1978), previous work with single xenograft lines suggests that the concentration of antigen in these models varies little from tumour to tumour (Munjal & Goldenberg, 1976; Chao *et al.*, 1974; Miwa *et al.*, 1977, 1976)—a 4-fold variation being the highest quoted (Carrel *et al.*, 1976). It remains to be seen whether different methods of xenografting and maintaining xenograft lines produce tumours of differing stabilities with respect to antigen content.

It has become apparent that the level of circulating CEA found in patients with colorectal carcinoma is a function not only of tumour size, but also of many other features of tumour and host biology (Goldenberg *et al.*, 1976*a*). The proportion of a tumour's cell population producing and excreting CEA clearly plays an important role. From *in vitro* studies, the highest cellular content and maximum production of CEA by colorectal-carcinoma cells is seen in the stationary or G1 phase of the cell cycle, whereas, conversely, exponentially growing cell populations have relatively low CEA content. However, release of CEA is not necessarily related to cell-cycle phase (Drewinko & Yang, 1976, 1980; Cohen & Wood, 1979). Tumour CEA concentration and total CEA burden will therefore be heavily dependent upon the kinetics of cell cycling within individual tumours, although the rate of CEA release need not necessarily be proportional to tumour CEA concentration and total CEA burden. It has also been suggested that CEA may be released during or after cell death (Bivins *et al.*, 1975; Davies & Steel, 1978; Breuer *et al.*, 1980); if so, the cell-loss rate of a tumour would also influence rate of CEA release. It is reasonable to suggest that levels of circulating CEA will also be affected by the relative partitioning of released CEA between the tumour interstitium, blood vascular space and gut lumen (for primary tumours (Molnar *et al.*, 1976)). Not surprising, therefore, is the finding that the degree of vascularization (Bivins *et al.*, 1975; Sharkey *et al.*, 1977; Zamcheck *et al.*, 1975) and lymphatic invasion (Zamcheck *et al.*, 1975) of tumours can be loosely related to plasma CEA

levels. Finally, host metabolism and degradation of CEA will affect circulating levels. Clearance of circulating CEA is thought to be mediated primarily by the liver (Shuster *et al.*, 1973; Primus *et al.*, 1973*a*), possibly by the hepatocytes. Thus depressed hepatic function may maintain circulating CEA at abnormally high levels, and the relative importance of CEA derived from normal gastrointestinal-tract cells may be increased (review by Loewenstein & Zamcheck, 1977). Decreased plasma CEA clearance may also result from the presence of circulating immune complexes formed between CEA and autologous anti-CEA (Primus *et al.*, 1973*a*, 1974) at least in experimental systems, although the significance of this in the human is as yet undetermined.

Given this complex array of factors potentially influencing circulating CEA levels, it is perhaps not surprising that no simple relationship exists between serum CEA and tumour size, weight, CEA concentration or tumour CEA burden in our system. The xenografts used show marked individual variation in CEA content; the exact degree of vascular invasion and necrosis varies from tumour to tumour (as judged by histological examination of tumour sections) and other relevant features such as host response and cell-cycle kinetic factors may vary from tumour to tumour, though we have not yet examined these. This is not to say that xenografts are inappropriate models for the study of CEA metabolism. It may be necessary for future studies of circulating CEA, and its relationship to aspects of tumour biology, to assess such features sequentially in individual xenograft-bearing animals, rather than across a population of such tumours.

The authors are indebted to Mr F. Gowali for his skilled technical assistance, and to Dr P. A. Smith for his examination of histological material. The work was supported by the Cancer Research Campaign and the Medical Research Council.

### REFERENCES

Bivins, B. A., Meeker, W. R. & Griffen, W. O. (1975) Carcinoembryonic antigen (CEA) levels and tumour histology in colon cancer. *J. Surg. Res.*, **18**, 257.

Breuer, H., Wagener, C. & Mueller-Wallraf, R. (1980) Mechanisms of CEA release into plasma in gastric and colorectal cancer. In *Abstracts of 8th meeting of Int. Soc. Oncodevelopmental Biol. Med., Tallinn, Estonia, U.S.S.R.* p. 61.

Carrel, S., Sordat, B. & Merenda, C. (1976) Establishment of a cell line (Co-115) from a human colon carcinoma transplanted into nude mice. *Cancer Res.*, **36**, 3978.

Chao, H.-F., Peiper, S. C., Philpott, G. W., Parker, C. W. & Aach, R. D. (1974) Selective uptake of specifically purified antibodies to carcinoembryonic antigen of human adenocarcinoma. *Res. Commun. Chem. Pathol. Pharmacol.*, **9**, 749.

Cobb, L. M. (1973) The behaviour of carcinoma of the large bowel in man following transplantation into immune-deprived mice. *Br. J. Cancer*, **28**, 400.

Cohen, A. M. & Wood, W. C. (1979) Carcinoembryonic antigen release from colorectal cancer cells in tissue culture. *J. Surg. Res.*, **27**, 372.

Davies, A. J. S. & Steel, G. G. (1978) In *Review of human tumour xenografts.* London: MRC. p. 3.

Dhar, P., Moore, T., Zamcheck, N. & Kupchik, H. A. (1975) Carcinoembryonic antigen (CEA) in colonic cancer. *J. Am. Med. Assoc.*, **221**, 31.

Drewinko, B. & Yang, L.-Y. (1976) Restriction of CEA synthesis to the stationary phase of growth of cultured human colon carcinoma cells. *Exp. Cell. Res.*, **101**, 414.

Drewinko, B. & Yang, L.-Y. (1980) Observations on the synthesis of CEA by an established human colonic carcinoma cell line. *Oncology*, **37**, 89.

Dyce, B. J. & Haverback, B. J. (1974) Free and bound carcinoembryonic antigen in neoplasms and in normal adult and fetal tissue. *Immunochemistry*, **11**, 423.

Gallo, V., Golferini, A., Capussotti, R., Gubetta, L., Rizzetto, M. & Tassi, G. C. (1977) Localization of carcinoembryonic antigen (CEA) in normal, inflammatory and neoplastic colonic mucosa by immunofluorescence. *Boll. Ist. Sieroter. Milan*, **56**, 132.

Goldenberg, D. M., Pletsch, Q. A. & van Nagell, J. R. (1976*a*) Characterization and localization of carcinoembryonic antigen in a squamous cell carcinoma of the cervix. *Gynecol. Oncol.*, **4**, 204.

Goldenberg, D. M., Sharkey, R. M. & Primus, F. J. (1976*b*) Carcinoembryonic antigen in histopathology: Immunoperoxidase staining of conventional tissue sections. *J. Natl Cancer Inst.*, **57**, 11.

Greenwood, F. G., Hunter, W. M. & Glover, J. S. (1963) The preparation of $^{131}$I-labelled growth hormone of high specific radioactivity. *Biochem. J.*, **89**, 114.

Holyoke, D., Reynoso, G. & Chu, T. M. (1972) Carcinoembryonic antigen (CEA) in patients with carcinoma of the digestive tract. *Ann. Surg.*, **176**, 559.

Keep, P. A., Leake, B. A. & Rogers, G. T. (1978) Extraction of CEA from tumour tissue, foetal colon and patients' sera, and the effect of perchloric acid. *Br. J. Cancer*, **37**, 171.

Khoo, S. K., Warner, N. L., Lie, J. T. & Mackay, I. R. (1973) Carcinoembryonic antigenic activity of tissue extracts: A quantitative study of malignant and benign neoplasms, cirrhotic liver, normal adult and fetal organs. *Int. J. Cancer*, **11**, 681.

KUBOTA, T., SHIMOSATO, Y. & NAGAI, K. (1978) Experimental chemotherapy of carcinoma of the human stomach and colon serially transplanted in nude mice. *Gann*, **69**, 299.

LOEWENSTEIN, M. S. & ZAMCHECK, N. (1977) Carcinoembryonic antigen and the liver. *Gastroenterology*, **72**, 161.

LOONEY, W. B., MAYO, A. A., ALLEN, P. M., MORROW, J. Y. & MORRIS, H. P. (1973) A mathematical evaluation of tumour growth curves in rapid, intermediate and slow growing rat hepatoma. *Br. J. Cancer*, **27**, 341.

MACH, J. P., CARREL, S., MERENDA, C., SORDAT, B. & CEROTTINI, J.-C. (1974) *In vivo* localization of radiolabelled antibodies to carcinoembryonic antigen in human colon carcinoma grafted into nude mice. *Nature*, **248**, 704.

McCLENDON, J. E., APPLEBY, D., CLAUDON, D. B., DONEGAN, W. L. & DECOSSE, J. J. (1977) Colonic neoplasms; Tissue estrogen receptors and carcinoembryonic antigen. *Arch. Surg.*, **112**, 240.

MIWA, M., SAKURA, H., KAWACHI, T. & 5 others (1976) Serum carcinoembryonic antigen level and transplanted colonic tumour size in nude mice. In *Onco-developmental Gene Expression*. Ed. Fishman & Sell. New York: Academic Press. p. 423.

MIWA, M., HIROHASHI, S., SHIMOSATO, Y. & 5 others (1977) Coproduction of carcinoembryonic antigen and α-fetoprotein in transplantable human colon cancer in nude mice. In *Proceedings 2nd Int. Workshop on Nude Mice*. Ed. Nomura *et al.* Stuttgart: Gustav Fischer. p. 435.

MOLNAR, I. G., VANDEVOORDE, J. P. & GITNICK, G. L. (1976) Carcinoembryonic antigen levels in fluids bathing gastrointestinal tumours. *Gastroenterology*, **70**, 513.

MUNJAL, D. & GOLDENBERG, D. M. (1976) Partial purification and immunochemical characterization of the carcinoembryonic antigen in xenografted GW39 human colonic tumours. In *Onco-developmental Gene Expression*. Ed. Fishman & Sell. New York: Academic Press. p. 621.

PRIMUS, F. J., GOLDENBERG, D. M. & HANSEN, H. J. (1973*a*) Metabolism of carcinoembryonic antigen in a human tumor—hamster host model. *Fed. Proc.*, **32**, 834.

PRIMUS, F. J., HANSEN, H. J. & GOLDENBERG, D. M. (1974) Altered metabolism of carcinoembryonic antigen in hamsters bearing GW-39 tumours. *Nature*, **249**, 837.

PRIMUS, F. J., WANG, R. H., COHEN, E., HANSEN, H. J. & GOLDENBERG, D. M. (1976) Antibody to carcinoembryonic antigen in hamsters bearing GW-39 human tumours. *Cancer Res.*, **36**, 2176.

PRIMUS, F. J., WANG, R. H., HANSEN, H. J. & GOLDENBERG, D. M. (1973*b*) Characterization of antibody to carcinoembryonic antigen (CEA) in hamsters xenografted with a human colonic tumor. *Proc. Am. Assoc. Cancer Res.*, **14**, 105.

RAGHAVAN, D., GIBBS, J., NOGUEIRA COSTA, R. & 4 others (1980) The interpretation of marker protein assays: A critical appraisal in clinical studies and a xenograft model. *Br. J. Cancer*, **41**, 191.

REYNOSO, G., CHU, T. M., HOLYOKE, D. & 6 others (1972) Carcinoembryonic antigen in patients with different cancers. *J. Am. Med. Assoc.*, **220**, 361.

SHARKEY, R. M., HAGIHARA, P. F. & GOLDENBERG, D. M. (1977) Localization by immunoperoxidase and estimation of radioimmunoassay of carcinoembryonic antigen in colonic polyps. *Br. J. Cancer*, **35**, 179.

SHUSTER, J., SILVERMAN, M. & GOLD, P. (1973) Metabolism of human carcinoembryonic antigen in xenogeneic animals. *Cancer Res.*, **33**, 65.

STRAGAND, J. J., YANG, L.-Y. & DREWINKO, B. (1980) Serum CEA levels in a human colonic adenocarcinoma (LoVo) xenograft system. *Cancer Lett.*, **10**, 45.

WAGENER, C. & BREUER, H. (1978) Comparative studies on the radioimmunological determination of carcinoembryonic antigen in tumour tissue. *J. Clin. Chem. Clin. Biochem.*, **16**, 323.

WARNER, N. L., KHOO, S. K., MACSWEEN, J. M., BANKHUSRT, A. D. & MACKAY, I. P. (1973) A micro-radioimmunoassay for carcinoembryonic antigen in whole serum and tissues. In *Host Interactions in the Etiology of Cancer in Man*. Ed. Zamcheck. Lyons: I.A.R.C. Sci. Publ. p. 317.

ZAMCHECK, N., DOOS, W. G., PRUDENTE, R., LURIE, R. B. & GOTTLIEB, L. S. (1975) Prognostic factors in colon carcinoma. *Hum. Pathol.*, **6**, 31.


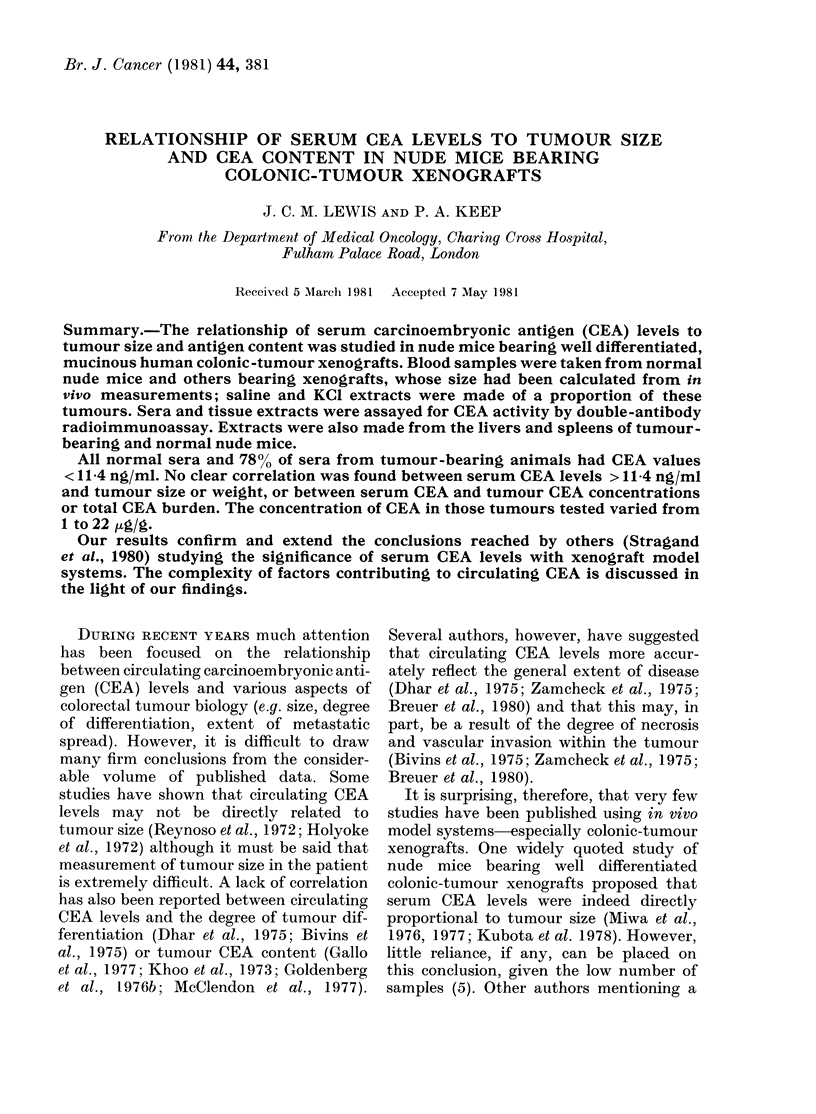

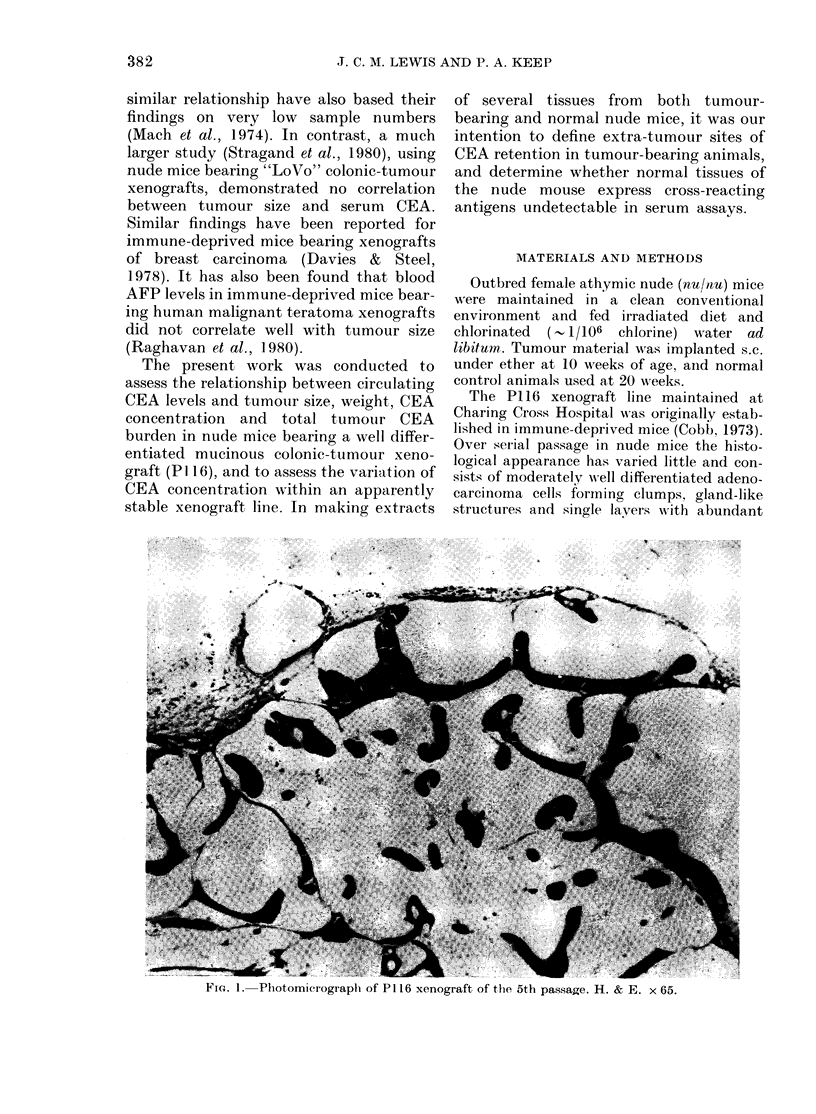

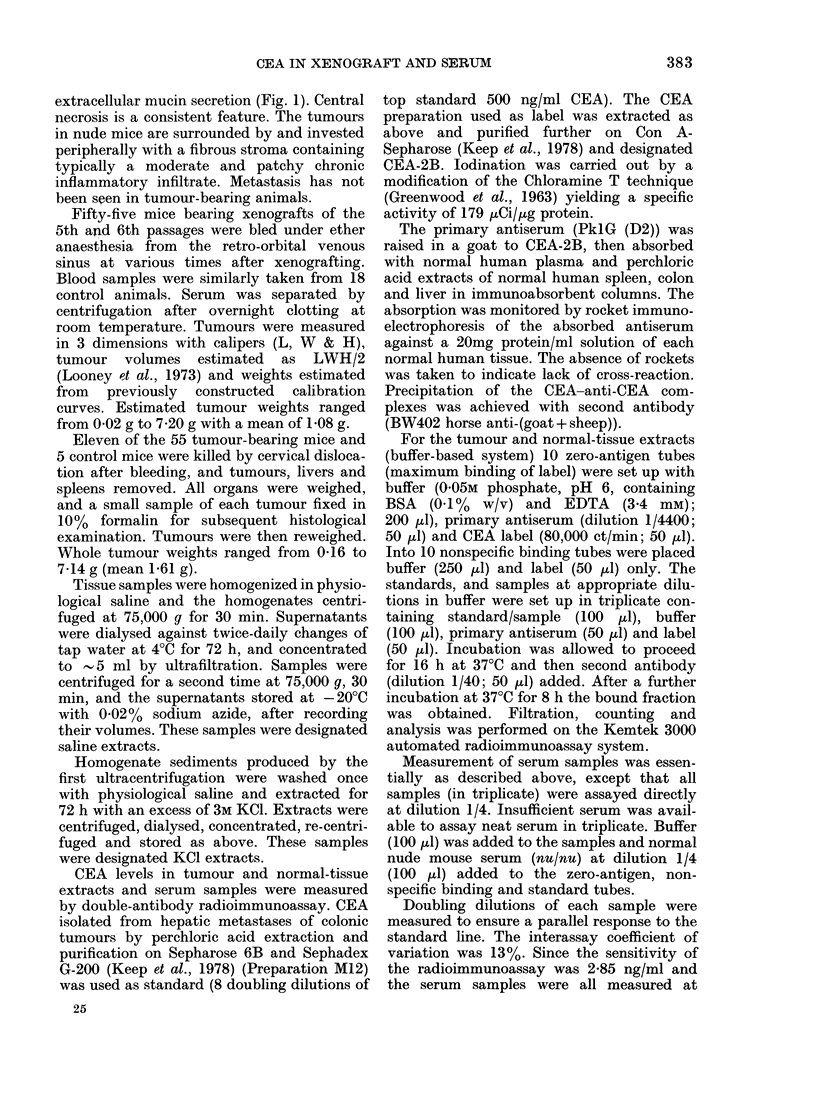

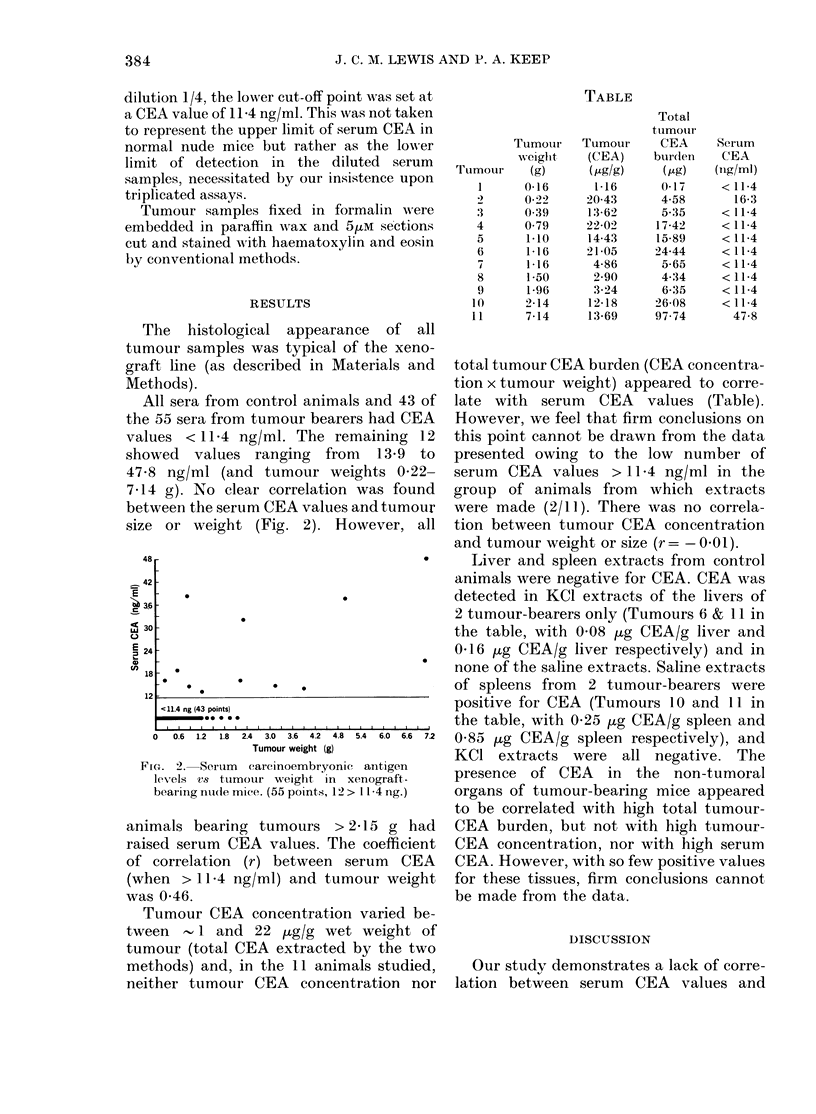

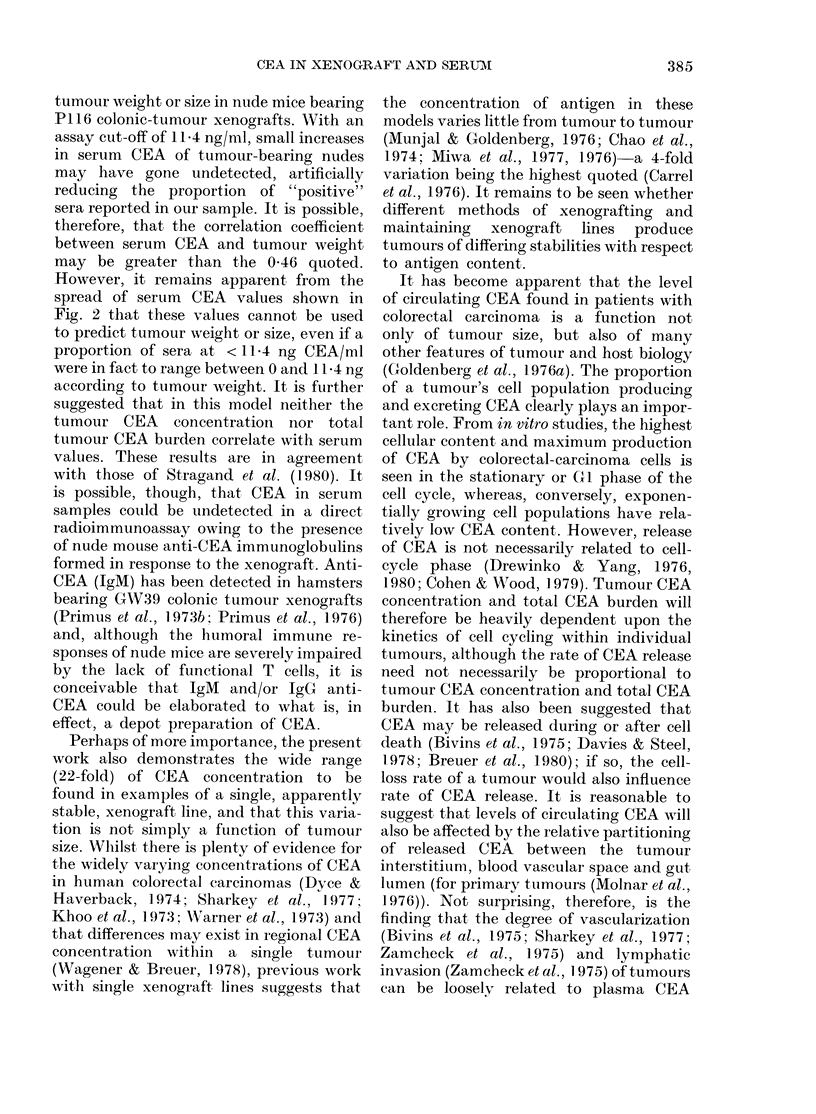

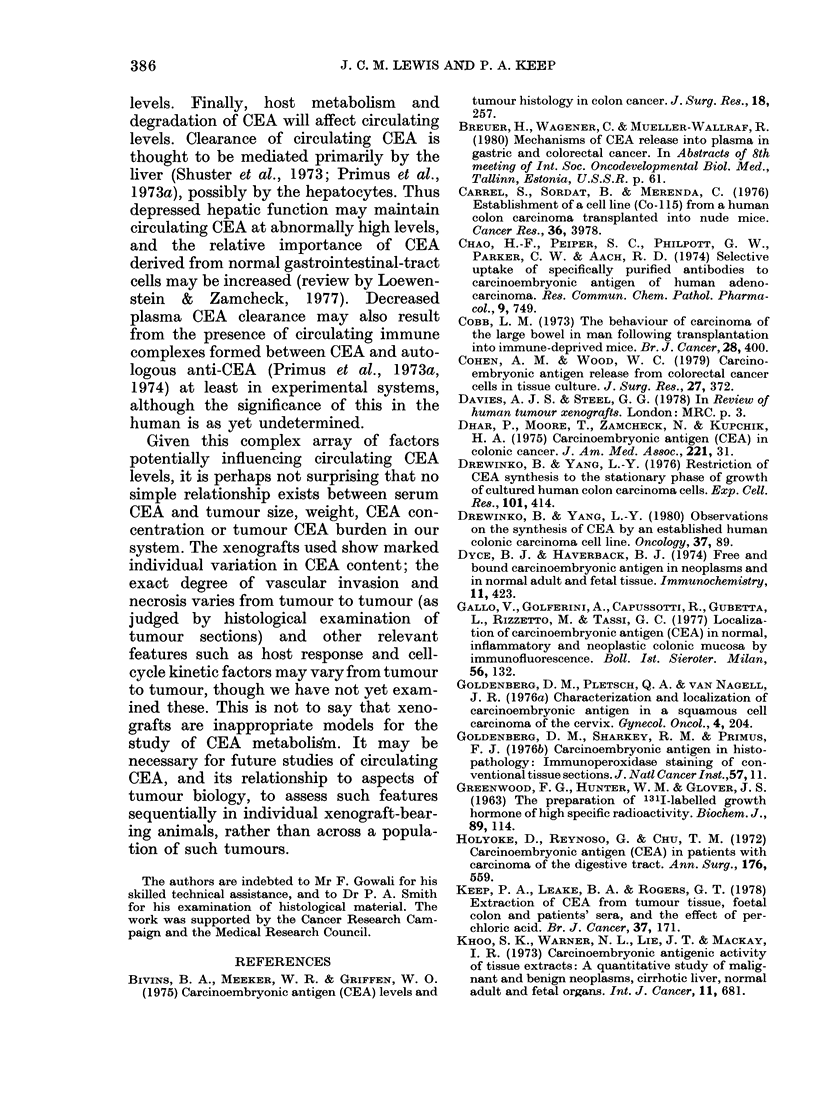

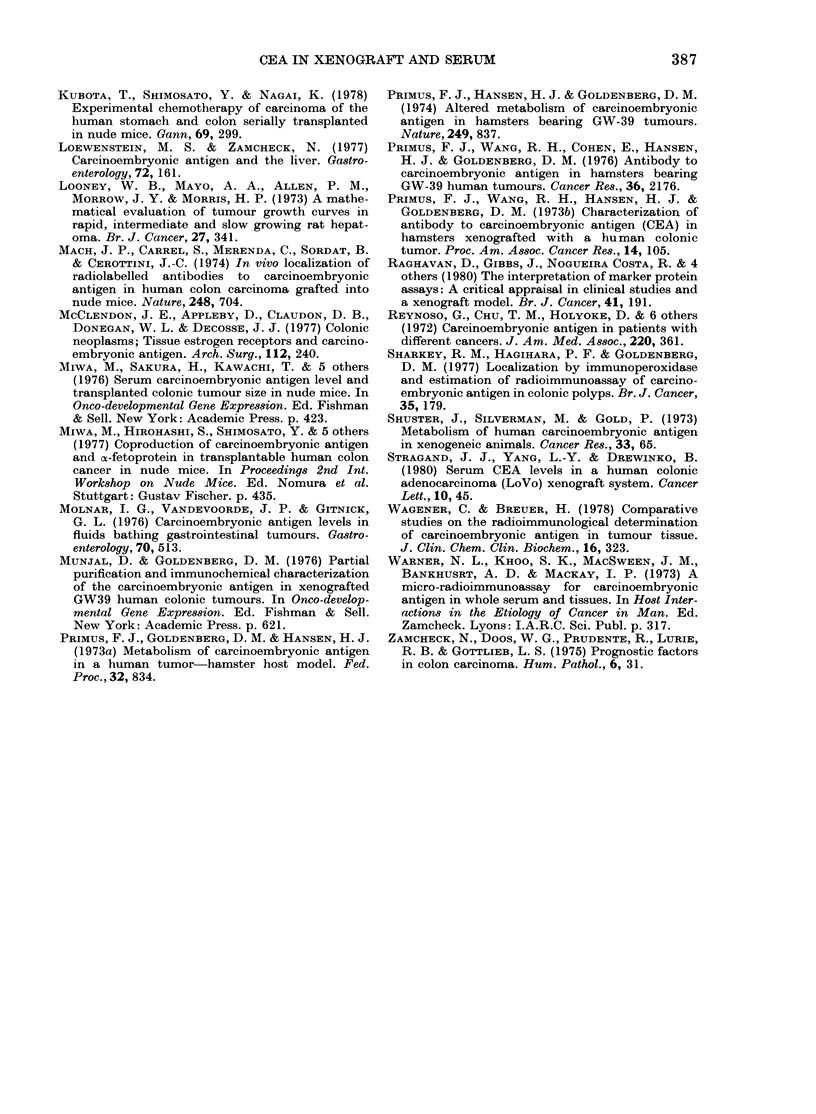


## References

[OCR_00610] Bivins B. A., Meeker W. R., Griffen W. O., Pellegrini J., Parker J. C. (1975). Carcinoembryonic antigen (CEA) levels and tumor histology in colon cancer.. J Surg Res.

[OCR_00624] Carrel S., Sordat B., Merenda C. (1976). Establishment of a cell line (Co-115) from a human colon carcinoma transplanted into nude mice.. Cancer Res.

[OCR_00630] Chao H., Peiper S. C., Philpott G. W., Parker C. W., Aach R. D. (1974). Selective uptake of specifically purified antibodies to carcinoembryonic antigen of human adenocarcinoma.. Res Commun Chem Pathol Pharmacol.

[OCR_00638] Cobb L. M. (1973). The behaviour of carcinoma of the large bowel in man following transplantation into immune deprived mice.. Br J Cancer.

[OCR_00642] Cohen A. M., Wood W. C. (1979). Carcinoembryonic antigen release from colorectal cancer cells in tissue culture.. J Surg Res.

[OCR_00651] Dhar P., Moore T., Zamcheck N., Kupchik H. Z. (1972). Carcinoembryonic antigen (CEA) in colonic cancer. Use in preoperative and postoperative diagnosis and prognosis.. JAMA.

[OCR_00656] Drewinko B., Yand L. Y. (1976). Restriction of CEA synthesis to the stationary phase of growth of cultured human colon carcinoma cells.. Exp Cell Res.

[OCR_00662] Drewinko B., Yang L. Y. (1980). Observations on the synthesis of carcinoembryonic antigen by an established human colonic carcinoma cell line.. Oncology.

[OCR_00667] Dyce B. J., Haverback B. J. (1974). Free and bound carcinoembryonic antigen in neoplasms and in normal adult and fetal tissue.. Immunochemistry.

[OCR_00692] GREENWOOD F. C., HUNTER W. M., GLOVER J. S. (1963). THE PREPARATION OF I-131-LABELLED HUMAN GROWTH HORMONE OF HIGH SPECIFIC RADIOACTIVITY.. Biochem J.

[OCR_00673] Gallo V., Golferini A., Capussotti R., Gubetta L., Rizzetto M., Tassi G. C. (1977). Localization of carcinoembryogenic antigen (CEA) in normal, inflammatory and neoplastic colonic mucosa by immunofluorescence.. Boll Ist Sieroter Milan.

[OCR_00681] Goldenberg D. M., Pletsch Q. A., Van Nagell J. R. (1976). Characterization and localization of carcinoembryonic antigen in a suqauamous cell carcinoma of the cervix.. Gynecol Oncol.

[OCR_00687] Goldenberg D. M., Sharkey R. M., Primus F. J. (1976). Carcinoembryonic antigen in histopathology: immunoperoxidase staining of conventional tissue sections.. J Natl Cancer Inst.

[OCR_00698] Holyoke D., Reynoso G., Chu T. M. (1972). Carcinoembryonic antigen (CEA) in patients with carcinoma of the digestive tract.. Ann Surg.

[OCR_00808] Jones B. R. (1957). OCULAR SYMPTOMS IN GLANDULAR FEVER.. Br J Ophthalmol.

[OCR_00704] Keep P. A., Leake B. A., Rogers G. T. (1978). Extraction of CEA from tumour tissue, foetal colon and patients' sera, and the effect of perchloric acid.. Br J Cancer.

[OCR_00710] Khoo S. K., Warner N. L., Lie J. T., Mackay I. R. (1973). Carcinoembryonic antigenic activity of tissue extracts: a quantitative study of malignant and benign neoplasms, cirrhotic liver, normal adult and fetal organs.. Int J Cancer.

[OCR_00719] Kubota T., Shimosato Y., Nagai K. (1978). Experimental chemotherapy of carcinoma of the human stomach and colon serially transplanted in nude mice.. Gan.

[OCR_00725] Loewenstein M. S., Zamcheck N. (1977). Carcinoembryonic antigen and the liver.. Gastroenterology.

[OCR_00730] Looney W. B., Mayo A. A., Allen P. M., Morrow J. Y., Morris H. P. (1973). A mathematical evaluation of tumour growth curves in rapid, intermediate and slow growing rat hepatomata.. Br J Cancer.

[OCR_00737] Mach J. P., Carrel S., Merenda C., Sordat B., Cerottini J. C. (1974). In vivo localisation of radiolabelled antibodies to carcinoembryonic antigen in human colon carcinoma grafted into nude mice.. Nature.

[OCR_00744] McClendon J. E., Appleby D., Claudon D. B., Donegan W. L., DeCosse J. J. (1977). Colonic neoplasms: tissue estrogen receptor and carcinoembryonic antigen.. Arch Surg.

[OCR_00765] Molnar I. G., Vandevoorde J. P., Gitnick G. L. (1976). CEA levels in fluids bathing gastrointestinal tumors.. Gastroenterology.

[OCR_00785] Primus F. J., Hansen H. J., Goldenberg D. M. (1974). Altered metabolism of carcinoembryonic antigen in hamsters bearing GW-39 tumours.. Nature.

[OCR_00791] Primus F. J., Wang R. H., Cohen E., Hansen H. J., Goldenberg D. M. (1976). Antibody to carcinoembryonic antigen in hamsters bearing GW-39 human tumors.. Cancer Res.

[OCR_00804] Raghavan D., Gibbs J., Nogueira Costa R., Kohn J., Orr A. H., Barrett A., Peckham M. J. (1980). The interpretation of marker protein assays: a critical appraisal in clinical studies and a xenograft model.. Br J Cancer Suppl.

[OCR_00810] Reynoso G., Chu T. M., Holyoke D., Cohen E., Nemoto T., Wang J. J., Chuang J., Guinan P., Murphy G. P. (1972). Carcinoembryonic antigen in patients with different cancers.. JAMA.

[OCR_00815] Sharkey R. M., Hagihara P. F., Goldenberg D. M. (1977). Localization by immunoperoxidase and estimation by radioimmunoassay of carcinoembryonic antigen in colonic polyps.. Br J Cancer.

[OCR_00822] Shuster J., Silverman M., Gold P. (1973). Metabolism of human carcinoembryonic antigen in xenogeneic animals.. Cancer Res.

[OCR_00827] Stragand J. J., Yang L. Y., Drewinko B. (1980). Serum CEA levels in a human colonic adenocarcinoma (LOVO) xenograft system.. Cancer Lett.

[OCR_00833] Wagener C., Breuer H. (1978). Comparative studies on the radioimmunological determination of carcinoembryonic antigen in tumour tissue.. J Clin Chem Clin Biochem.

[OCR_00847] Zamcheck N., Doos W. G., Prudente R., Lurie B. B., Gottlieb L. S. (1975). Prognostic factors in colon carcinoma: correlation of serum carcinoembryonic antigen level and tumor histopathology.. Hum Pathol.

